# Color Vision in Schoolchildren with Low Birth Weight and Those Born Full-Term with Appropriate Weight for Gestational Age

**DOI:** 10.3390/vision9030070

**Published:** 2025-08-12

**Authors:** Paula Yuri Sacai, Maria Cecília Saccomani Lapa, Rosana Fiorini Puccini, Nívea Nunes Ferraz

**Affiliations:** 1 Departamento de Oftalmologia e Ciências Visuais, Escola Paulista de Medicina, Universidade Federal de São Paulo—Unifesp, São Paulo 04023-062, Brazil; psacai@unifesp.br (P.Y.S.);; 2 Departamento de Pediatria, Escola Paulista de Medicina, Universidade Federal de São Paulo—Unifesp, São Paulo 04023-062, Brazil; rpuccini@unifesp.br

**Keywords:** color vision, low birth weight, schoolchildren, dyschromatopsia, Farnsworth D-15

## Abstract

Purpose: To evaluate color discrimination in schoolchildren with low birth weight (LBW) and those born full-term and at a weight appropriate for gestational age (AGA). Methods: LBW children aged 5–11 years and school-, grade-, sex-, and age-matched full-term (birth weight ≥ 2500 g) AGA controls from 14 randomly selected schools from a low-income region were tested. Examinations included visual acuity, ocular motility, and color vision testing using the Farnsworth D-15 test. Color score and interocular color score difference (ICD) were compared between the groups. Multiple logistic regression was used to analyze associations between color vision deficit and group, adjusting for age, sex, visual acuity, strabismus, and amblyopia. Results: A total of 291 LBW children (age = 8.5 ± 1.3 yrs; 55.7% females) and 265 AGA children (age = 8.5 ± 1.4 yrs; 56.2% females) were examined. Dyschromatopsia was detected in 10.3% of LBW and 7.9% of AGA children, primarily involving tritan and non-specific defects. Color scores were comparable between the groups, and color deficit was significantly associated with younger age and worse visual acuity. The ICD was statistically larger (*p* = 0.004) in the LBW group, in which the frequencies of strabismus and amblyopia were also higher. Conclusions: Most LBW children demonstrated normal color discrimination, but their interocular color score difference was larger than that of AGA children.

## 1. Introduction

The World Health Organization defines low birth weight (LBW) as birth weight inferior to 2500 g, which may be caused by inadequate intra-uterine growth and/or preterm birth (gestational age below 37 weeks), and is generally associated with adverse socioeconomic conditions [1]. In Brazil, LBW affects around 8% of the born-alive infants who are therefore subject to chronic or neurological diseases, possible disabling sequelae, learning difficulties, behavioral disturbances, and cognitive alterations, such as language, hearing, and/or vision deficits [2]. Visual dysfunctions in infancy may impact the child’s daily activities, social and family living, self-esteem, and quality of life [3]. Reports in the literature show that LBW is frequently related to prematurity and the retinopathy of prematurity. But even having no neurological or ocular structural disorders, LBW children commonly present refractive errors, strabismus, and functional visual deficits, which may vary from mild to severe visual impairment and affect visual acuity, stereopsis, contrast sensitivity, and color vision [4,5].

Color vision is an important function of the visual system, as colors enrich the perception of the environment and facilitate object discrimination [6,7]. This is a fascinating area of science, challenging the arts, industry, and those who try to understand the physiological mechanisms of both chromatic sensation and interpretation. Color is a cerebral interpretation based on the comparison of objects’ spectral reflectances influenced by the genes that encode the photopigments and the cognitive aspects that modulate this perception [8]. The extensive use of color code in educational and professional scenarios makes the clinical evaluation of this visual function relevant [6,7]. Recognizing and naming colors are important abilities that play a significant role in a child’s development and learning. Thus, the early detection of color vision deficits can help parents and teachers adopt strategies that enhance learning for tasks requiring chromatic discrimination during childhood [6,7]. However, in some countries, neither clinical ophthalmological assessments nor ocular health programs include the routine evaluation of color vision. Consequently, subtle color visual dysfunctions may not be detected and possibly affect the schoolchildren’s daily activities, impacting professional development in adulthood [5,7,9,10].

Color vision deficiency, or dyschromatopsia, is characterized by an impaired ability to differentiate specific colors. Dyschromatopsia can be either congenital or acquired, and is classified according to the confusion axis, which is the area on a color plot where an individual cannot distinguish colors adequately [11]. Deuteranomaly is the most common type of color vision deficiency, affecting especially the middle-wavelength sensitive (green) cones. Protanomaly primarily affects the long-wavelength (red) cones, and tritanomaly affects the short-wavelength (blue) cones [11]. The congenital form manifests as a red–green axis deficit (protan and/or deutan defects) and is more prevalent in the male population, affecting up to 8% of European men [12,13,14] and 6.5% of Asian men [15,16,17,18,19]. Blue–yellow axis defects (tritan defect) are typically acquired and have a significantly higher prevalence in preterm children, being over 200 times more common than in the general adult population [20].

The studies on color vision in LBW children are scarce, and available reports involve samples of premature newborns with retinopathy of prematurity [5,20,21,22]. Color defects (particularly tritan) in this population have been attributed to the retinal damage resulting from increased ambient lighting in the neonatal unit [20,22]. Moreover, visual function in full-term LBW children remains an underexplored topic in the literature. Therefore, this study aimed to evaluate color discrimination in a group of preterm and full-term schoolchildren with LBW and those born full-term and at a weight appropriate for gestational age (AGA).

## 2. Materials and Methods

This observational descriptive transversal study is part of the research project entitled “Morbidity, growth and development of low-birth-weight schoolchildren aged 6 to 10 child’s care integrity and intersectorality in the local health system of Embu das Artes, Sao Paulo”. The project was carried out under the tenets of the Declaration of Helsinki and was approved (protocol code 1142/09, 16 October 2009) by the Research Ethics Committee of the Federal University of Sao Paulo (Unifesp).

### 2.1. Participants

This study included schoolchildren with low birth weight (LBW < 2500 g at birth, regardless of gestational age) and those born full-term and at a weight appropriate for gestational age (AGA ≥ 2500 g), all aged 5–11 years and enrolled in municipal public schools in a low-income region of Brazil.

According to the local Municipal Secretariat of Education, 17,326 schoolchildren enrolled in municipal schools met the stated criteria. Out of these, 788 weighed below 2500 g at birth and attended the schools that were randomly selected for the study. The parents/caregivers of the identified LBW schoolchildren were contacted by school authorities, were notified of the study purposes, and were invited to sign the informed consent form, allowing an evaluation of their children’s vision at school. The children who wore spectacles were requested to bring them on the examination day. At the same schools, the same steps were applied to create the control group (AGA), which included schoolchildren who had an appropriate weight for gestational age, and they were matched by school, grade, sex, and age.

Sample size requirements were calculated based on estimating an expected color vision deficit prevalence of 6% [20] within an error bound (precision) of 3% with a 95% confidence interval, adjusting for the finite population factor (considering the population of 788 LBW children), which resulted in a minimum required sample size of 185 LBW schoolchildren.

### 2.2. Procedures

The exams were performed during school hours and included visual acuity measurement, ocular motility assessment, and color discrimination testing by a trained team.

Presenting visual acuity (VA) was determined for each eye (starting from the right eye) using a tumbling-E optotypes logMAR chart for 40 cm. Ocular motility was evaluated for near and distance vision using the cover test and the Irvine–Jampolsky (four diopters) test.

Color discrimination was assessed monocularly (starting from the right eye) at 50 cm by the Farnsworth Dichotomous Panel D-15 test (Good-Lite, Elgin, IL, USA). The standard illumination was assured by an artificial daylight source at 6500 K. The test consists of a clear plastic case with a reference disk at a fixed location and 15 saturated colored disks in plastic caps, which subtend a visual angle of 1.5° at 50 cm. The D15 disks represent the hue colors of the natural color system/circle and are similar in saturation to those of the Farnsworth–Munsell 100 Hue test. The movable caps are numbered on the back and make up an incomplete color circle. The subjects were instructed to order the 15 color disks, choosing a disk that most closely matched the color of the previous reference disk. The test was performed only once, but at the end, children were allowed to rearrange the caps one time if necessary. The participants completed the D-15 test in both eyes in about 10 min.

### 2.3. Data Management and Analysis

**Figure 1:**
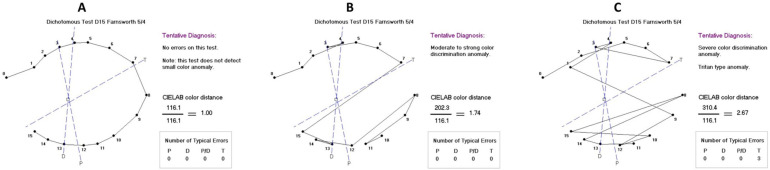
Representative graphs of color discrimination and respective chromatic classification obtained by the Farnsworth Dichotomous Panel D-15 test. (**A**) Normal color discrimination; (**B**) Moderate color deficit; (**C**) Severe color deficit.

The Farnsworth Dichotomous Panel D-15 scoring software (Visual Mill 1.0, Color Vision Evaluation) was used to evaluate participants’ color vision discrimination, providing error score and axis. For the analysis, the color discrimination was categorized as normal (error score ranging from 1.00 to 1.68), moderate (1.69 to 2.24), or severe deficit (from 2.25 on), according to the chromatic classification results (type and error number) provided by the software (Box 1 and Figure 1A–C). Types of dyschromatopsia were also analyzed, including the following defects: deutan, protan, tritan, red-green, and monochromacy. Cases classified by the software as undetermined color defects were considered non-specific.

Box 1 **Color score and chromatic classification provided by the Visual Mill Program.**

**Chromatic Classification**

**Score Range**

**Color Discrimination Category**

**min**

**max**
Normal Range.1.001.25NormalModerate color discrimination weakness.1.251.67Moderate color discrimination weakness.1.271.65Moderate color discrimination weakness. Possible tritan type anomaly.1.191.68Moderate color discrimination weakness. Possible red–green type anomaly.1.391.62Moderate to strong color discrimination anomaly. Tritan type anomaly.1.692.05Moderate deficitModerate to strong color discrimination anomaly. Deutan type anomaly.1.761.76Moderate to strong color discrimination anomaly. Red–green type anomaly.1.721.72Moderate to strong color discrimination anomaly. Non-specific anomaly.1.712.21Strong color discrimination anomaly. Non-specific anomaly.2.292.49Severe deficitStrong color discrimination anomaly. Tritan type anomaly.2.302.51Strong color discrimination anomaly. Deutan type anomaly.2.322.32Strong color discrimination anomaly. Red–green type anomaly.2.362.38Severe color discrimination anomaly. Tritan type anomaly.2.742.93Severe color discrimination anomaly. Deutan type anomaly.2.942.94Severe color discrimination anomaly. Red–green type anomaly.2.963.17Severe color discrimination anomaly. Protan type anomaly.3.503.50Severe color discrimination anomaly. Extreme trichromat or monochromat.2.813.64

The results were compared between the groups (LBW versus AGA) and analyzed using the color score obtained from the better color discrimination eye and the interocular color score difference (ICD). Moreover, the color score results were investigated for children with very low birth weight (VLBW < 1500 g). A multiple logistic regression model was performed to analyze the association between color deficit (score ≥ 1.69) and group, adjusting for age, sex, visual acuity, presence of strabismus, and amblyopia. The influence of birth weight, strabismus, and interocular acuity on the ICD was investigated by multiple linear regression. Stata Statistical Software Release 14.0 (StataCorp LLC, College Station, TX, USA) was used for statistical analyses, and *p* ≤ 0.05 was considered significant.

## 3. Results

The study group consisted of 291 LBW schoolchildren (162 females—55.7%) selected from 14 randomly selected public schools, with ages ranging from 5.9 to 10.7 years (mean = 8.5 ± 1.3 years; median = 8.7 years). Birth weights varied from 620 to 2490 g (mean = 2084 ± 404 g; median = 2230 g), given that 28 children had a very low birth weight (mean = 1125 ± 226 g; median = 1127 g). Out of all, 123 (42.3%) participants were premature (range 22–36 weeks), 145 (49.8%) were born at term (range 37–41 weeks), and gestational age information was not available for 23 (7.9%).

The AGA group included 265 children (149 females—56.2%) aged 5.0 to 11.8 years (mean = 8.5 ± 1.4 years; median = 8.7 years) with a birth weight of ≥2500 g (mean = 3266 ± 456 g; median = 3200 g).

Mean visual acuity in the better-seeing eye was 0.07 ± 0.17 logMAR (Snellen equivalent 20/24) in the LBW group and 0.04 ± 0.12 logMAR (20/25) in the AGA group, given that thirty-four participants of the total sample wore glasses (13 LBW and 21 AGA). Visual acuity was statistically better in the AGA group (Mann–Whitney z = −2.460, *p* = 0.014), but the proportion of glasses users was comparable between both groups (Chi2 = 2.89, *p* = 0.089). In addition, the frequencies of strabismus (33 LBW and 12 AGA; Chi2 = 8.65, *p* = 0.003) and amblyopia (15 LBW and 3 AGA; Chi2 = 7.16, *p* = 0.007) were statistically higher in the LBW group.

According to color score classification, out of 556 schoolchildren, 90.8% (261 LBW and 244 AGA) presented normal color discrimination. A moderate deficit was found in 5.2% (16 LBW and 13 AGA; 55.2% female), and a severe deficit in 4.0% (14 LBW and 8 AGA; 50.0% female) of the participants. Dyschromatopsias were identified in 51 schoolchildren (30 LBW and 21 AGA), affecting girls (*n* = 27) and boys (*n* = 24) in a similar proportion (Chi2 = 0.20, *p* = 0.651), as well as participants of LBW (*n* = 30) and AGA (*n* = 20) groups (Chi2 = 0.95, *p* = 0.331). Data by group and sex are presented in Table 1. Blue–yellow/tritan (11 LBW and 7 AGAs; Chi2 = 0.06, *p* = 0.806) and non-specific color defects (13 LBW and 7 AGA; Chi2 = 0.52, *p* = 0.472) were the main findings, detected in, respectively, 3.2% and 3.6% of the sample, being more frequent in the LBW group, but without statistical significance. Red–green/deutan/protan defects were found in 1.4% of the participants (four LBW and four AGA).

The mean color score was 1.28 ± 0.40 (range 1.00–3.64; median = 1.15) for the LBW group and 1.26 ± 0.42 (range 1.00–3.61; median = 1.11) for the AGA group, with no statistical difference (z = −0.61, *p* = 0.55). In multiple logistic regression modeling (Table 2), color deficit was significantly associated with younger age (OR 0.30 [95% CI 0.14–0.63]) and worse visual acuity (OR 2.56 [95% CI 1.60–4.08]). Children aged 9 years and older had a 70% lower chance of color deficits. For each octave (three lines in logMAR chart) of worsening visual acuity, the chance of color deficit increased 2.56 times, adjusted for other variables. No interactions were observed between birth weight, sex, strabismus, or amblyopia and color score deficit.

LBW children (mean = 0.26 ± 0.29; median = 0.17) presented a mean ICD that was statistically larger (z = −2.09, *p* = 0.04) than the AGA (mean = 0.19 ± 0.21; median = 0.13). Multiple linear regression showed that ICD was significantly associated with group (OR 0.05 [95% CI 0.01–0.09; *p* = 0.017]) and interocular acuity difference (OR 0.28 [95% CI 0.10–0.46; *p* = 0.003]). The expected ICD increase was 0.05 in LBW children and 0.3 for each line of interocular acuity difference, adjusted for the other variables.

For the 28 children who had a very low birth weight, the mean color score was 1.36 ± 0.50 and the mean ICD was 0.31 ± 0.37. When comparing these participants to the other 263 LBW children, no statistically significant differences were found for either variable. Additionally, premature participants showed similar color vision results (score, ICD, deficit level, and dyschromatopsia frequency) to the full-term schoolchildren in the LBW group.

## 4. Discussion

LBW children may develop several visual dysfunctions. In general, these conditions are associated with very low birth weight (≤1500 g) and/or prematurity (gestational age < 26 weeks), as a consequence of retinopathy of prematurity or lesions in the posterior visual pathways affecting the central or peripheral nervous system [4,21,23]. From another perspective, this study evaluated a large sample of LBW children attending mainstream schooling, matched by school, grade, sex, and age with children born full-term and appropriate weight for gestational age.

The vast majority of our sample, 90% of LBW children and 92% of AGA children, demonstrated normal color discrimination, consistent with prior studies [5,7,20]. Concerning color deficit findings, only 10% (*n* = 30) of our LBW sample presented some dyschromatopsia, with non-specific color defect being the main alteration, representing 43% (*n* = 13) of the cases, followed by tritan defect, found in 37% (*n* = 11). In accordance with the literature, our results indicate that dyschromatopsias are not related to low birth weight [5,21,22], since the non-specific (*n* = 7) and tritan (*n* = 7) color defects were also observed in the AGA group, in which 8% (*n* = 21) of participants presented some form of dyschromatopsia. In the LBW group, half of the dyschromatopsia cases were premature children, of which the majority showed a tritan defect (*n* = 6). A previous study on preterm children identified the tritan defect as the most frequent form of dyschromatopsia [22], associated with retinal structural damage [20,22]. It is essential to note that the D-15 may not be sensitive to detect congenital color deficits linked to X chromosome as protan/deutan dyschromatopsias, since this test is a screening tool and does not favor a full characterization of color anomalies [21,24].

Another important consideration regarding the Farnsworth D-15 is that the attention and memory mechanisms, as well as cognitive processes, may influence test performance because motor, verbal, and intellectual competencies were required from the child during the assessment [25,26,27]. It is worth highlighting that our study methodology included monocular color vision evaluation, which may cause the second eye tested to yield either a worse result due to fatigue or a lack of interest, or a better result due to the learning effect. In our study, each of these two situations was observed in a similar proportion of the schoolchildren with normal/near normal visual acuity (VA of 0.3 logMAR or better) and without interocular acuity difference; 14% (*n* = 63) of the participants presented the same color score in both eyes, 44% (*n* = 206) had better color score in the first eye tested, and 42% (*n* = 194) had better color score in the second eye tested. The fatigue or learning effect may explain our results of younger children (in both LBW and AGA groups), who had a higher chance of worse color discrimination, maybe owing to cognitive immaturity, as the visual maturation process is age-dependent [26,28]. Furthermore, our children with visual acuity reduction, regardless of group, had a higher chance of worse color discrimination, which is corroborated by previous studies that pointed out deficits in color vision in the presence of severe visual acuity impairment [29,30].

The literature reports that strabismus and refractive errors are common ocular findings in LBW children [4,5,21]. In our study, the frequencies of strabismus and amblyopia were, respectively, three and five times higher in the LBW children, and their interocular color score difference was also significantly larger. Moreover, our results showed that interocular color score difference was significantly associated with low birth weight and interocular acuity difference, and it could pass unnoticed in basic clinical investigations [5,10].

A limitation of the current work is that the medical records of the children who underwent perinatal hospitalization were not accessed, which means that the presence of underlying neuropediatric conditions cannot be ruled out in this population, hindering the correlations of the findings of this study. In addition, no assessments were conducted to identify neuropsychomotor and learning disorders, which could impact the child’s performance on visual tests. Another consideration is the school environment used for visual evaluation, which is more likely to lead to children’s attention wandering during the tests compared to an outpatient healthcare setting. Additionally, detailed ocular motility, refraction, and fundus exams were not performed.

Despite the lack of a complete ophthalmological assessment, the current study included a large sample size, made possible by school-based sampling, which is logistically less cumbersome and costly. Also, this study included a matched control group of AGA children, with a similar number of participants, sociodemographic characteristics, and living conditions as the LBW group. Thus, our results of color discrimination and visual acuity reflect the daily visual condition of a group of schoolchildren from public schools in a low-income region of Brazil, among whom 9% presented some form of dyschromatopsia. These and other subtle color visual dysfunctions not detected in childhood may impact school activities and learning, as well as professional activities involving color vision in adulthood.

Future studies on color visual function in children using tests for detecting congenital and acquired color deficits may contribute to a better understanding of color dysfunctions in childhood. Additionally, research is necessary to determine the frequency, causes, risk factors, and types of strabismus, amblyopia, and refractive errors in children with low birth weights.

## 5. Conclusions

Normal color discrimination was found in most of the LBW and AGA children who were attending mainstream schooling. Dyschromatopsias were identified in 9% of the total sample, being mainly tritan and non-specific color defects.

The interocular color score difference and the frequencies of strabismus and amblyopia were higher in the LBW group than in the AGA group.

These data can contribute to pediatric ophthalmology clinical practice, encouraging clinical investigation and the understanding of color deficits and their influence on children’s visual performance in daily life and school. Additionally, this study may support the medical guidance and education of parents, caregivers, and teachers regarding visual deficits in children, which may affect global development and learning.

## Figures and Tables

**Table 1:** Types of dyschromatopsia according to sex and group.

Dyschromatopsia	Male (*n*)		Female (*n*)	
	LBW	AGA	LBW	AGA
Monochromacy	2	1	0	2
Red–green	3	0	0	2
Deuteranomaly	1	1	0	0
Protanomaly	0	0	0	1
Tritanomaly	3	3	8	4
Non-specific	8	2	5	5
Total	17	7	13	14

*n*: number of participants, LBW: low-birth-weight group, AGA: wight appropriate for gestational age group.

**Table 2:** Odds ratios and 95% confidence intervals in multivariate analysis of factors related to color deficit.

Variable	OR [95% CI]	*p* Value
Group		
AGA	reference	
LBW	1.11 [0.60–2.06]	0.753
Age range (years)		
<9	reference	
≥9	0.30 [0.14–0.63]	0.002 *
Sex		
Female	reference	
Male	1.22 [0.66–2.24]	0.525
Visual acuity		
Octave	2.56 [1.60–4.08]	<0.001 *
Strabismus		
No	reference	
Yes	1.21 [0.41–3.56]	0.726
Amblyopia		
No	reference	
Yes	1.21 [0.27–5.46]	0.808

OR: odds ratio; CI: confidence interval; * *p* < 0.01.

## Data Availability

The original contributions presented in this study are included in the article. Further inquiries can be directed to the corresponding author.

## Abbreviations

The following abbreviations are used in this manuscript:

LBW	Low birth weight
AGA	Appropriate weight for gestational age
ICD	Interocular color score difference
